# Inhibition of adjuvant-induced arthritis by nasal administration of novel synthetic peptides from heat shock protein 65

**DOI:** 10.1186/1471-2474-15-253

**Published:** 2014-07-25

**Authors:** Xiao-Lian Shi, Li-Ping Wang, Xuan Feng, Dan-Dan Fan, Wei-Jin Zang, Bing Wang, Jun Zhou

**Affiliations:** 1 Department of Pharmacology, Xi’an Jiaotong University Health Science Center, Xi’an, Shaanxi 710061, P.R. China; 2 Department of Pathology and Therapeutic Vaccines Engineering Center, Xi’an Jiaotong University Health Science Center, Xi’an, Shaanxi 710061, P.R. China

**Keywords:** Adjuvant arthritis, Synthetic peptides, Inflammatory cytokines, Heat shock protein

## Abstract

**Background:**

Rheumatoid arthritis (RA) is a chronic systemic inflammatory disease mediated by T cells. The aim of the present study was to investigate the therapeutic efficacy of synthetic peptides (HP-R1, HP-R2 and HP-R3), derived from the sequence of 65-kD mycobacterial heat shock protein (HSP), in the treatment of RA using adjuvant-induced arthritis (AA) animal model.

**Methods:**

AA was induced by a single intradermal injection Freund’s complete adjuvant in male Lewis rats. At the first clinical sign of disease, rats were administered nasally by micropipette of peptides or phosphate buffer saline (PBS). Disease progression was monitored by measurement of body weight, arthritis score and paw swelling. The changes of histopathology were assessed by hematoxylin eosin staining. The serum levels of tumor necrosis factor (TNF) - alpha and interleukin (IL)-4 were measured by enzyme-linked immunosorbent assay (ELISA).

**Results:**

The peptides efficiently inhibited the footpad swelling and arthritic symptoms in AA rats. The synthetic peptides displayed significantly less inflammatory cellular infiltration and synovium hyperplasia than model controls. This effect was associated with a suppression of pro-inflammatory cytokine TNF-alpha production and an increase of anti-inflammatory cytokine IL-4 production after peptides treatment.

**Conclusions:**

These results suggest that the synthetic peptides derived from HSP65 induce highly effective protection against AA, which is mediated in part by down-regulation of inflammatory cytokines, and support the view that the synthetic peptides is a potential therapy for RA that may help to diminish both joint inflammation and destruction.

## Background

Rheumatoid arthritis (RA) is a systemic autoimmune disease, characterized by the presence of inflammatory synovitis and destruction of joint cartilage and bone. This disease leads to severe pain and reduces life expectancy. The underlying immunologic mechanisms and etiologic factors responsible for the initiation and development of RA are not yet fully understood, but it is well established that inflammation and bone destruction plays crucial roles in RA. RA is specifically characterized by enhanced production of tumor necrosis factor (TNF)-alpha, interferon-gamma (IFN) - gamma, interleukin (IL)-6 and IL-17, and in the affected joints [1-3]. Therefore, therapeutic agents which possess anti-inflammatory and immunosuppressant activity will provide important benefits and promises in the treatment of RA. Non-steroidal anti-inflammatory drugs (NSAIDs) such as indomethacin and celecoxib, represent an effective therapy for treating RA. Disappointingly, this kind of medicine has side-effects including gastric ulcer and the cardiovascular risk, which has limited its use [4-6]. Therefore, there is still an unmet need for improved therapies for RA.

Heat shock proteins (HSPs) are ubiquitous housekeeping proteins found in virtually all living organisms. HSPs have been a subject of expanding interest in human and experimentally induced autoimmune diseases, both as potential antigens and as intracellular chaperones involved in peptide binding to human leucocyte antigens. Previous studies have reported that nasal administration of mycobacterial HSP60 peptide induces highly effective protection against adjuvant-induced arthritis (AA), an experimental arthritis model with close histopathologic resemblance to RA [7,8]. Notably, our recent study has demonstrated that the synthetic peptides from HSP65 inhibit pro-inflammatory cytokine secretion by peripheral blood mononuclear cells from RA patients [9].

However, there is no scientific evidence to prove the effectiveness of the synthetic peptides from HSP65 on RA in the vivo model. In the present study, we employed AA rat model and treated the rats with Heat shock protein- derived peptide (HP). To investigate the therapeutic effect of these peptides, we detected arthritis severity, footpad swelling, and anti-inflammatory effects of these peptides.

## Methods

### Animals

Male inbred Lewis rats (6–9 weeks old) were obtained from the Vital River Laboratory Animal Technology Co. Ltd. (Beijing, China). Rats were maintained under climate-controlled conditions under 12-h dark–light cycle and had unlimited access to water and standard rat chow. All experimental procedures were performed in accordance with the Guidelines for the Care and Use of Laboratory Animals issued by the Chinese Council on Animal Research and the Guidelines of Animal Care. This study was approved by the ethics committee of Xi’an Jiaotong University.

### Materials

Freund’s complete adjuvant (FCA) was obtained from sigma (St. Louis, MO, USA). Three peptides used in this study were identified by bioinformatics tools and comprised dominant epitopes recognized by T cells. The peptides are protected by a patent (CN 200810150035.8). Three 15-mer peptides (HP-R1, QKRAAQDAAVDAACG; HP-R2, QKRAAQAARVEAACG; and HP-R3, QKLFKTLQSLFADFN) were synthesized by Chengdu Kaijie Biomedical Technology Development (Chengdu, China). The purity of the HP-R1, HP-R2 and HP-R3 peptides was 99.13%, 96.84% and 98.14%, respectively. All three peptides had good antigenicity and immunogenicity.

### Adjuvant induced arthritis and peptide immunotherapy protocols

Rats were lightly anesthetized with ether and adjuvant arthritis (AA) was induced by a single intradermal (i.d.) injection in the base of the tail with 150 μl FCA composed of heat-killed and dried Mycobacterium tuberculosis (strain H37Ra, ATCC 25177) suspended in paraffin oil and mannide monooleate. Rats were inspected daily for the onset of arthritis as characterized by edema and erythema in the paws. Peptide was administered on day 10, 13, 16, and 19 after induction of arthritis with FCA. Briefly, 100 μg of peptide dissolved in phosphate buffer saline (PBS) (peptide concentration 10 mg/ml) was administered nasally in a total volume of 10 μl (5 μl per nostril) using a micropipette. An equal volume of PBS was given rats in control group and AA model group. Every group consists of 9 animals [10].

### Assessment of arthritis

Arthritis disease progression and severity were evaluated by arthritic clinical scoring, measurement of hind paw volumes and body weight for every two days following induction of arthritis in a blinded manner. Hind paw volumes were observed by rat paw volume measurement instrument. The level of arthritic inflammation of each paw was graded from 0 to 4 based on degree of swelling, erythema and deformation of the joints: 0 = normal; 1 = slight erythema or swelling of one of the toe or fingers; 2 = erythema and swelling of two toes or fingers; 3 = severe erythema and swelling of the ankle or wrist; 4 = complete erythema and swelling of toes or fingers, joint deformity and lack of flexibility. Thus the maximum score was 16 [11].

### Biochemical studies

On day 28 after the induction of arthritis the rats were sacrificed by CO_2_ inhalation, after which blood samples and hind limb joints were collected. Blood samples were centrifuged at 1500 g for 10 min at 4°C and serum was aliquoted and stored at −80°C until assay. Cytokines were measured by using enzyme-linked immunosorbent assay (ELISA). Briefly, the concentrations of inflammatory cytokine TNF-alpha and IL-4 in serum were quantified by using commercial cytokine ELISA kit (R&D systems, Minneapolis, USA) and performed in duplicate according to the manufacturer’s instructions.

### Histopathological studies

Hind limb joints were collected on day 28 after the induction of arthritis, after the rats were sacrificed by CO_2_ inhalation. The ankle joints were fixed in 10% buffered formalin for 24 h and decalcified in 10% ethylene diamine tetraacetate (EDTA) solution. After decalcification, tissues were dehydrated, processed, and embedded in paraffin wax. 5 μm serial sections from each block were prepared, stained with haematoxylin and eosin (H&E) and observed under a light microscopy.

### Statistical analysis

Data are presented as the mean ± SEM. Statistical analysis was performed by one-way analysis of variance followed by Tukey post-hoc test for multiple comparisons. The significant level was set at a two-tailed *P* < 0.05. All figures were prepared with GRAPHPAD PRISM 5.0 (San Diego, CA, USA).

## Results

### Effects of peptides on body weight of AA rats

**Figure 1:**
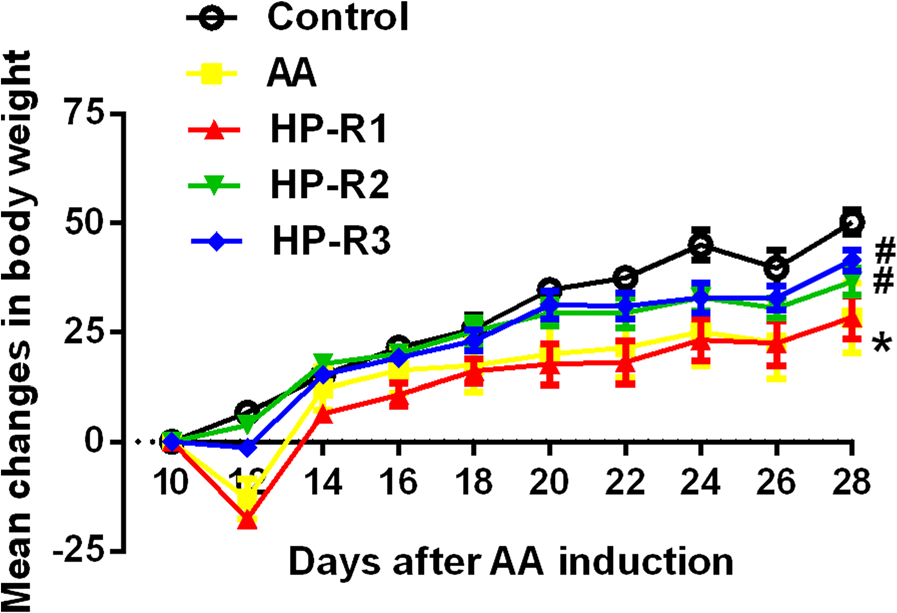
**Effect of HP-R1, HP-R2 and HP-R3 on body weight of AA rats.** Adjuvant Arthritis was induced on day 0 with Freund’s Complete Adjuvant (FCA). 100 μg peptides were administered nasally by micropipette on day 10, 13, 16, and 19 after FCA injection. An equal volume of PBS was given rats in control group and AA model group. Starting on day 8, the rats were weighed every two days. Data are presented as mean ± SEM, n =9 rats per group. *P < 0.05 when compared to control group, ^#^*P* < 0.05 when compared to AA group.

Body weight of the rats in control group growed steadily. The body weight of AA rats after FCA injection declined sharply, followed by slow growth. Peptides HP-R2 and HP-R3 significantly inhibited the decrease (*P* < 0.05) in body weight of AA rats on day 12 following FCA injection, compared to AA model group. The AA rats exhibited significant body weight gain (*P* < 0.05) in HP-R3 group; while the HP-R1 and HP-R2 also increased the body weight of AA rats, there was no significant difference (Figure 1).

### Effect of peptides on arthritic score of AA rats

**Figure 2:**
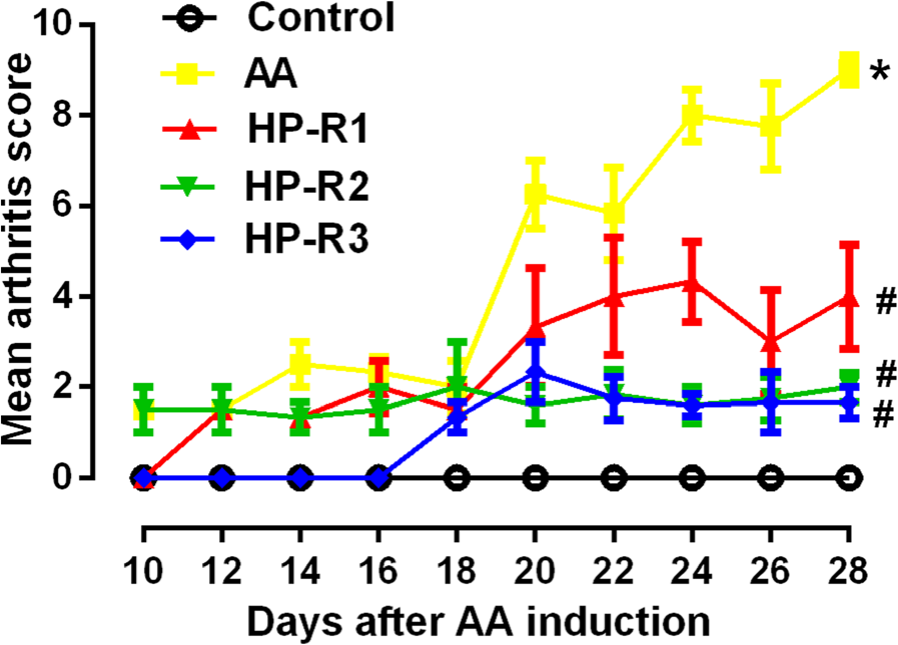
**Effect of HP-R1, HP-R2 and HP-R3 on arthritic score of AA rats.** Adjuvant Arthritis was induced on day 0 with Freund’s Complete Adjuvant (FCA). 100 μg peptides were administered nasally by micropipette on day 10, 13, 16, and 19 after FCA injection. An equal volume of PBS was given rats in control group and AA model group. Arthritis score was assessed every other day from day 8 onward. Data are presented as mean ± SEM, n =9 rats per group.

The arthritic index represents the grade of arthritis that was used to assess efficacy of three peptides. In the AA group, diseased rats without any treatment showed an increased arthritic index starting on day 10 to a peak on day 28. Compared with AA group, administration of HP-R1 significantly (*P* < 0.05) reduced arthritis score on day 22, 26, 28 and 34. HP-R2 and HP-R3 were observed to have significantly strong activity in preventing progression of arthritic disease, and arthritis score of AA rats were found to be significantly lowered (*P* < 0.05) from day 20 to day 34 (Figure 2).

### Effect of peptides on paw swelling of AA rats

**Figure 3:**
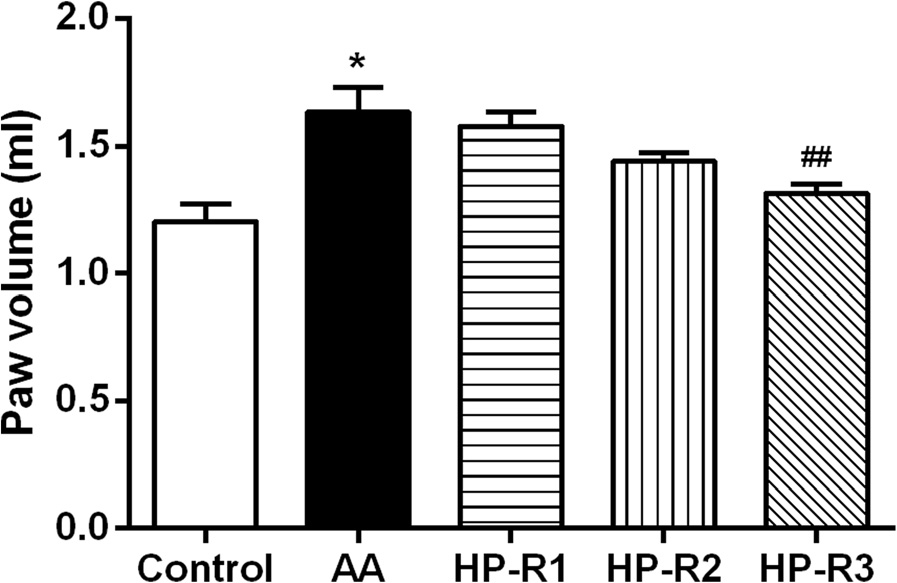
**Effect of HP-R1, HP-R2 and HP-R3 on paw swelling of AA rats.** Adjuvant Arthritis was induced on day 0 with Freund’s Complete Adjuvant (FCA). 100 μg peptides were administered nasally by micropipette on day 10, 13, 16, and 19 after FCA injection. An equal volume of PBS was given rats in control group and AA model group. Paw swelling was assessed when arthritis score reached a maximum on day 28. Data are presented as mean ± SEM, n =9 rats per group. ^*^P < 0.05 when compared to control group, ^##^*P* < 0.01 when compared to AA group.

The paw swelling in AA rat model also represents the severity of arthritis. We showed the paw swelling of rats when arthritis score was reached to a maximum on day 28. Compared with control group, paw swelling of the rats increased significantly in AA model group (*P* < 0.05). Although HP-R1 and HP-R2 tended to inhibit footpad swelling, the difference did not reach statistical significance. HP-R3 owed significant inhibitory activity in paw swelling compared to AA model group (*P* < 0.01). (Figure 3).

### Effect of peptides on biochemical parameters

**Figure 4:**
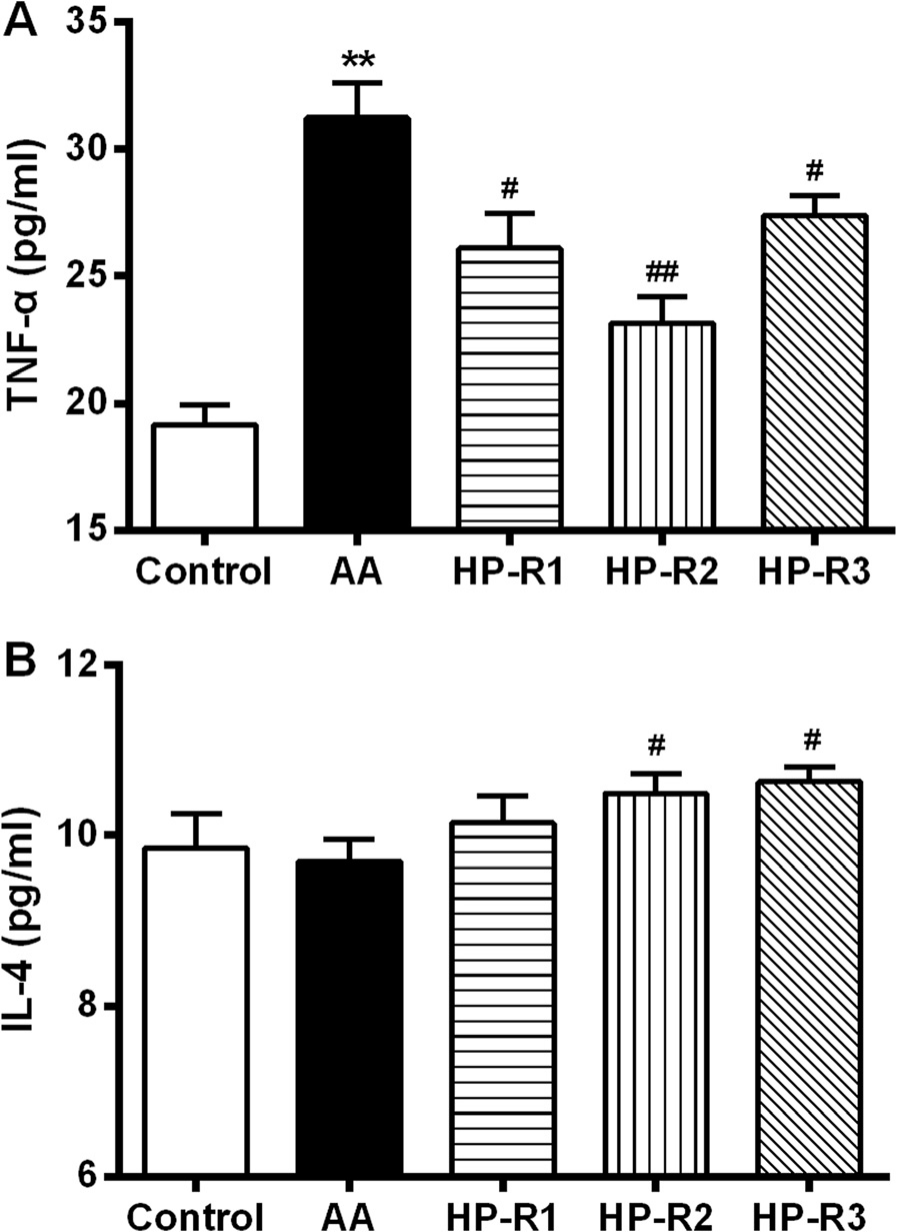
**Effect of HP-R1, HP-R2 and HP-R3 on the levels of TNF-alpha (A) and IL-4 (B) in serum.** Serum of rats was collected on day 28 after FCA injection and measured with ELISA kits. Data are presented as mean ± SEM, n = 9 rats per group. ^**^P < 0.01 when compared to control group, ^#^*P* < 0.05, ^##^*P* < 0.01 when compared to AA group.

Effect of three peptide on biochemical parameters was examined in AA rats by measuring serum cytokine (TNF-alpha and IL-4) levels on day 34 after adjuvant injection. Compared to the lower serum cytokine levels found in control rats, serum TNF-alpha levels were significantly increased (*P* < 0.01) in AA rats (Figure 4A). Treatment of AA rats with three peptides resulted in a significant decrease in serum TNF-alpha level as compared to the high serum TNF-alpha levels found in AA rats. Notably, anti-inflammatory cytokine IL-4 levels were significantly (*P* < 0.05) increased in HP-R2 and HP-R3 treated group (Figure 4B).

### Effect of peptides on histopathology of arthritic paws

**Figure 5:**
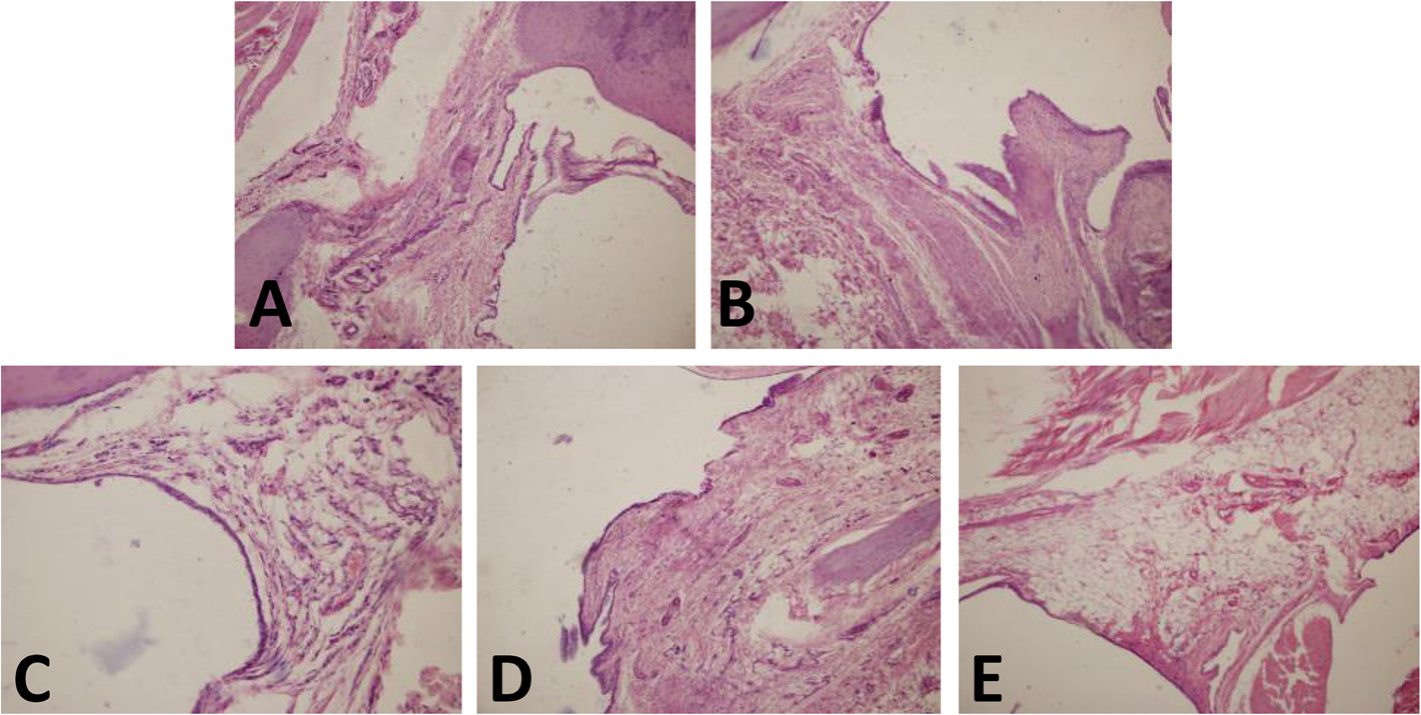
**Effects of HP-R1, HP-R2 and HP-R3 on histopathology of arthritic paws. A**: Control group; **B**: AA model group; **C**: AA rats administered with HP-R1; **D**: HP-R2; **E**: HP-R3. (H&E staining, magnification 10×). n = 4 per treatment group.

**Figure 6:**
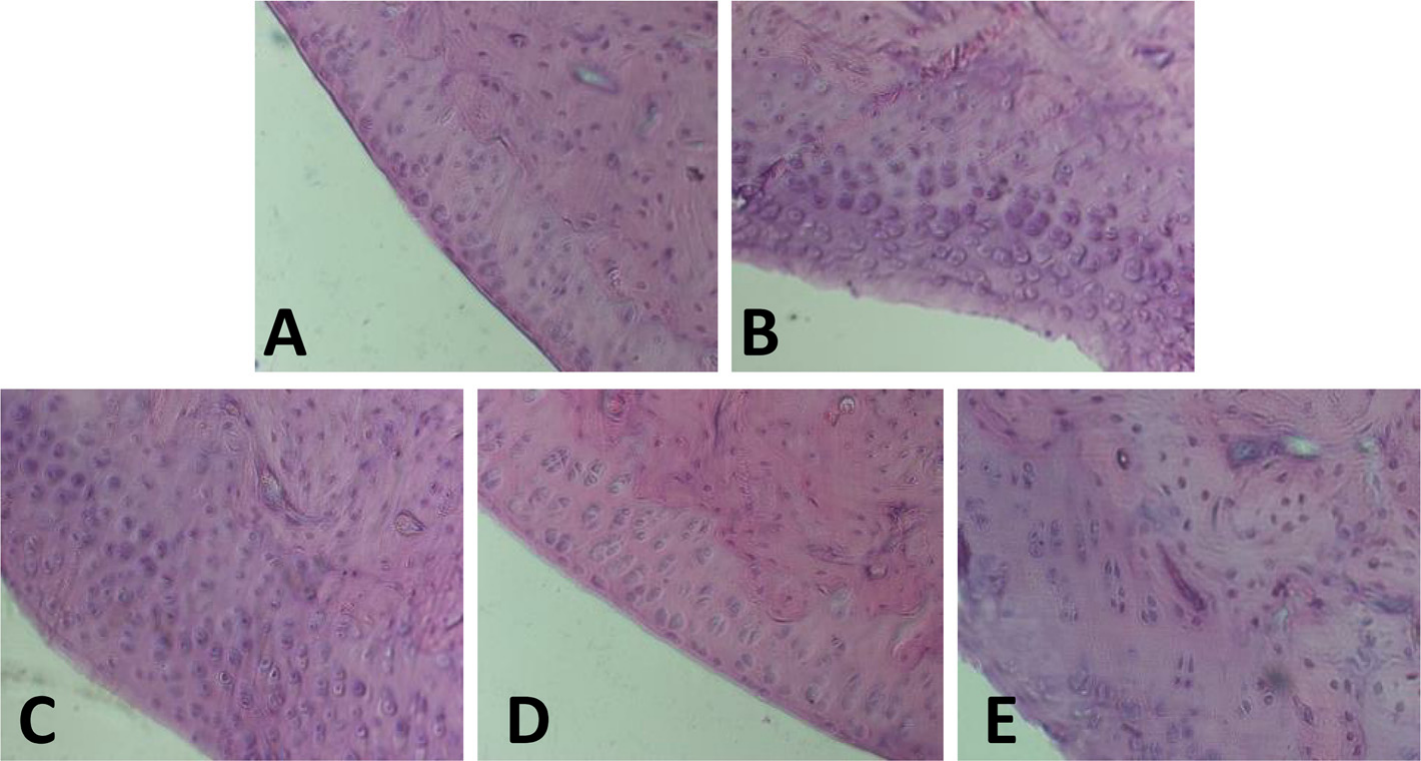
**Effects of HP-R1, HP-R2 and HP-R3 on cartilage cells of AA rats. A**: Control group; **B**: AA model group; **C**: AA rats administered with HP-R1; **D**: HP-R2; **E**: HP-R3. (H&E staining, magnification 10×). n = 4 per treatment group.

Histological analysis revealed that no synovial hyperplasia, inflammatory cell infiltration or tissue destruction were seen in control group by H&E staining (Figure 5A). In contrast, the ankle joint of AA rats in model group showed prominent synovial hyperplasia, inflammatory cellular infiltration and severe pannus formation (Figure 5B). Compared with AA model group, cellular infiltration and synovium hyperplasia were markedly reduced in the rats treated with three peptides (Figure 5C-E).In addition, we further observed the changes in cartilage cells of AA rats. The results in control group showed smooth articular cartilage surface and cartilage cells arranged in neat rows (Figure 6A). In contrast, AA model group revealed that there were rough cartilage surface, small cracks, a small amount of clumping of the cartilage cells, and significantly increased cartilage cells (Figure 6B). However, cartilage cells were markedly reduced and smooth cartilage surface was also observed in the rats treated with three peptides compared with AA model group (Figure 6C-E).

## Discussion

RA is characterized by synovial inflammation, destruction of cartilage and bone, severe joint pain, and ultimately life-long crippling. In the present study, the synthetic peptides derived from HSP65 were studied for their efficacy on RA treatment employing an adjuvant-induced arthritis rat model, which is extensively used for studies regarding the pathogenesis of autoimmune arthritis as well as for the validation of the efficacy of anti-rheumatic drugs because it has various similarities to human RA [12-14]. Administrations of three peptides from the day of disease onset significantly inhibited the arthritic clinical scoring and paw swelling. We also observed dampening effects on TNF-alpha concomitant with dramatic increases in IL-4 production in the serum after peptides treatment, which was relevant to inhibiting arthritic progression. These findings were positively consistent with the results of histological examinations, which reduced cellular infiltration and synovium hyperplasia in the peptide-treated groups.

The disease onset in rats commenced from day 10 and reached maximum on day 28 in the adjuvant induced (disease control) arthritic rats. The synthetic peptides from HSP65 efficiently inhibited the progression of AA with a significant reduction of the clinical score. The most important index for evaluation of arthritis is the footpad swelling, and these peptides showed the ability to inhibit the paw swelling of AA rats. Inflammatory cellular infiltration and prominent synovial hyperplasia were observed in H&E tissue sections of arthritic control animals. In contrast, the histopathological changes were restored to normal stage after giving treatment with three peptides.

Pro-inflammatory cytokines TNF-alpha, IL-1bata, and IFN-gamma produced by macrophages and other immune cells are of decisive importance in the pathogenesis and progression of RA [1-3]. Among the T cell, the T-helper (Th1) cells secrete TNF- alpha and IFN-gamma, whereas the counter-regulatory Th2 cells secrete IL-4 and IL-10 [7]. The role of Th1-Th2 balance plays an important role in regulation of autoimmunity [15,16]. In view of the critical role of pro-inflammatory cytokines in RA, scientists intend to design drugs for suppression of this disease via anti-inflammation. There is substantial evidence demonstrating the importance of TNF-alpha in the inflammatory process of RA [9,17], and this cytokine is better value to assess the degree of the arthritis and also good targets for treating the disease. Drugs that block pro-inflammatory cytokines TNF-alpha can improve outcomes for RA [18]. However, the anticytokine biologics are costly in the treatment of RA [19]. In the current study, the serum levels of TNF-alpha in arthritic rats were dramatically up-regulated as compared to control rats and these levels were markedly reduced after treatment with the synthetic peptides. Of note, HP-R2 and HP-R3 also increased levels of IL-4, resulting in an overall anti-inflammatory effect. IL-4 has been identified as a crucial anti-inflammatory cytokine in RA [20-23]. It is important to detect other cytokines such as IFN-gamma and IL-10. Our recent study has demonstrated that the secretion of IFN-gamma by peripheral blood mononuclear cells from RA patients was significantly suppressed by these synthetic peptides [9]. The peptides tended to increase IL-10, although the effect did not reach statistical significance. It was suggested that these peptides conferred the ability in treatment of RA via the inhibition of inflammation response.

It has been shown that disease-protective peripheral T cell tolerance can be induced through oral or nasal administration of an antigen [8]. Successful experiments in RA rat models have shown promising results using HSP peptides to modulate the arthritis disease [8,24,25]. Based on previous studies [25,26] and our current results, the administration of HSP65 appears to generate suppressor or regulatory T cells that may down-regulate the autoimmune responses by the release of suppressor cytokines, and as a result, the inflammation is suppressed. Compared with orally induced tolerance, nasally induced tolerance has been shown to be equally or, in the case of a peptide antigen, even more effective in curbing experimental autoimmune diseases [8,27]. Similar to oral tolerance, three distinct mechanisms (e.g., clonal deletion, clonal anergy, and active suppression) are possibly operational in nasal induced tolerance [24,27].

Recent study has demonstrated that an altered peptide ligand derived from HSP60 induces the activation of T cells by modifying cell cycle phase’s distribution of CD4+ T cells from RA patients [26]. Thus, CD4+ T cells may be involved in the immunomodulatory effects elicited by the three HSP peptides in the present study. Further investigations are clearly required to detect the immunophenotype of the immune cells and elucidate which cells are HSP65 targets.

In conclusion, the results of the present study suggest that synthetic peptides containing a T cell-dominant epitope could efficiently inhibit the progression of AA with a significant reduction of the clinical and histopathogic score. This effect was associated with a suppression of TNF-alpha production and an increase of IL-4 production, which may indicate the presence of a Th2 type tolerogenic mechanism. The present study has led to the identification of a promising peptide candidate for RA treatment. Besides the possibility to use them as a therapeutic drug, they can be used as tools to define the antigen (epitope).

## Conclusion

These results suggest that the synthetic peptides derived from HSP65 induce highly effective protection against AA, which is mediated in part by down-regulation of inflammatory cytokines, and support the view that the synthetic peptides is a potential therapy for RA that may help to diminish both joint inflammation and destruction.

## Abbreviations

AA: Adjuvant-induced arthritis; EDTA: Ethylene diamine tetraacetate; ELISA: Enzyme-linked immunosorbent assay; FCA: Freund’s complete adjuvant; H&E: Haematoxylin and eosin; HP: Heat shock protein- derived peptide; HSP: Heat shock protein; IFN: Interferon; IL: Interleukin; NSAIDs: Non-steroidal anti-inflammatory drugs; PBS: Phosphate buffer saline; RA: Rheumatoid arthritis; Th: T-helper; TNF: Tumor necrosis factor.

## Competing interests

The authors declare that they have no competing interests.

## Authors’ contributions

JZ, XS and BW participated in research design. JZ, XS and LW conducted experiments. XS, XF and DF performed data analysis. JZ, XS and ZW wrote or contributed to the writing of the manuscript. JZ acquired funding for the research. All authors read and approved the final manuscript.

## Pre-publication history

The pre-publication history for this paper can be accessed here:

http://www.biomedcentral.com/1471-2474/15/253/prepub

## References

[B1] Hobl EL Mader RM Erlacher L Duhm B Mustak M Broll H Hogger P Kalipciyan M Jilma B The influence of methotrexate on the gene expression of the pro-inflammatory cytokine IL-12A in the therapy of rheumatoid arthritis Clin Exp Rheumatol 2011 29 6 963 969 22133036

[B2] McInnes IB Schett G Cytokines in the pathogenesis of rheumatoid arthritis Nat Rev Immunol 2007 7 6 429 442 17525752 10.1038/nri2094

[B3] Christodoulou C Choy EH Joint inflammation and cytokine inhibition in rheumatoid arthritis Clin Exp Med 2006 6 1 13 19 16550339 10.1007/s10238-006-0088-5

[B4] Rao P Knaus EE Evolution of nonsteroidal anti-inflammatory drugs (NSAIDs): cyclooxygenase (COX) inhibition and beyond J Pharm Pharm Sci 2008 11 2 81s 110s 19203472 10.18433/j3t886

[B5] White WB West CR Borer JS Gorelick PB Lavange L Pan SX Weiner E Verburg KM Risk of cardiovascular events in patients receiving celecoxib: a meta-analysis of randomized clinical trials Am J Cardiol 2007 99 1 91 98 17196469 10.1016/j.amjcard.2006.07.069

[B6] Zarraga IG Schwarz ER Coxibs and heart disease: what we have learned and what else we need to know J Am Coll Cardiol 2007 49 1 1 14 17207715 10.1016/j.jacc.2006.10.003

[B7] Venkatesha SH Rajaiah R Berman BM Moudgil KD Immunomodulation of autoimmune arthritis by herbal CAM Evid Based Complement Alternat Med 2011 2011 986797 21234398 10.1155/2011/986797 PMC3014691

[B8] Prakken BJ Roord S Van Kooten PJ Wagenaar JP Van Eden W Albani S Wauben MH Inhibition of adjuvant-induced arthritis by interleukin-10-driven regulatory cells induced via nasal administration of a peptide analog of an arthritis-related heat-shock protein 60 T cell epitope Arthritis Rheum 2002 46 7 1937 1946 12124879 10.1002/art.10366

[B9] Zhou J Wang LP Feng X Fan DD Zang WJ Wang B Synthetic peptides from heat-shock protein 65 inhibit proinflammatory cytokine secretion by peripheral blood mononuclear cells from rheumatoid arthritis patients Clin Exp Pharmacol Physiol 2014 41 1 67 72 24111596 10.1111/1440-1681.12178

[B10] Roord ST Zonneveld-Huijssoon E Le T Yung GP Koffeman E Ronaghy A Ghahramani N Lanza P Billetta R Prakken BJ Albani S Modulation of T cell function by combination of epitope specific and low dose anticytokine therapy controls autoimmune arthritis PLoS One 2006 1 e87 17183718 10.1371/journal.pone.0000087 PMC1762388

[B11] Naidu VG Dinesh BK Thwin MM Satish RL Kumar PV Gopalakrishnakone P RANKL targeted peptides inhibit osteoclastogenesis and attenuate adjuvant induced arthritis by inhibiting NF-kappaB activation and down regulating inflammatory cytokines Chem Biol Interact 2013 203 2 467 479 23333834 10.1016/j.cbi.2012.12.016

[B12] Rajaiah R Lee DY Ma Z Fan AY Lao L Fong HH Berman BM Moudgil KD Huo-Luo-Xiao-Ling Dan modulates antigen-directed immune response in adjuvant-induced inflammation J Ethnopharmacol 2009 123 1 40 44 19429337 10.1016/j.jep.2009.02.032 PMC2925191

[B13] Kim HR Rajaiah R Wu QL Satpute SR Tan MT Simon JE Berman BM Moudgil KD Green tea protects rats against autoimmune arthritis by modulating disease-related immune events J Nutr 2008 138 11 2111 2116 18936206 10.3945/jn.108.089912 PMC2693422

[B14] Durai M Kim HR Bala KK Moudgil KD T cells against the pathogenic and protective epitopes of heat-shock protein 65 are crossreactive and display functional similarity: novel aspect of regulation of autoimmune arthritis J Rheumatol 2007 34 11 2134 2143 17937454

[B15] Hussein YM El-Shal AS Rezk NA Abdel GS Alzahrani SS Influence of interleukin-4 gene polymorphisms and interleukin-4 serum level on susceptibility and severity of rheumatoid arthritis in Egyptian population Cytokine 2013 61 3 849 855 23394902 10.1016/j.cyto.2013.01.001

[B16] Dimitrova P Skapenko A Herrmann ML Schleyerbach R Kalden JR Schulze-Koops H Restriction of de novo pyrimidine biosynthesis inhibits Th1 cell activation and promotes Th2 cell differentiation J Immunol 2002 169 6 3392 3399 12218161 10.4049/jimmunol.169.6.3392

[B17] Meugnier E Coury F Tebib J Ferraro-Peyret C Rome S Bienvenu J Vidal H Sibilia J Fabien N Gene expression profiling in peripheral blood cells of patients with rheumatoid arthritis in response to anti-TNF-alpha treatments Physiol Genomics 2011 43 7 365 371 21266503 10.1152/physiolgenomics.00127.2010

[B18] Rifkin LM Birnbaum AD Goldstein DA TNF inhibition for ophthalmic indications: current status and outlook Biodrugs 2013 27 4 347 357 23568177 10.1007/s40259-013-0022-9

[B19] Page TH Brown A Timms EM Foxwell BM Ray KP Inhibitors of p38 suppress cytokine production in rheumatoid arthritis synovial membranes: does variable inhibition of interleukin-6 production limit effectiveness in vivo? Arthritis Rheum 2010 62 11 3221 3231 20589681 10.1002/art.27631

[B20] Balsa A Del AJ Blanco F Caliz R Silva L Sanmarti R Martinez FG Tejedor D Artieda M Pascual-Salcedo D Oreiro N Collado MD Andreu JL Graell E Simón L Martínez A Mulero J Prediction of functional impairment and remission in rheumatoid arthritis patients by biochemical variables and genetic polymorphisms Rheumatology (Oxford) 2010 49 3 458 466 20032229 10.1093/rheumatology/kep380

[B21] Szekanecz Z Koch AE Angiogenesis and its targeting in rheumatoid arthritis Vascul Pharmacol 2009 51 1 1 7 19217946 10.1016/j.vph.2009.02.002 PMC2917972

[B22] Tesmer LA Lundy SK Sarkar S Fox DA Th17 cells in human disease Immunol Rev 2008 223 87 113 18613831 10.1111/j.1600-065X.2008.00628.x PMC3299089

[B23] Gerli R Lunardi C Pitzalis C Unmasking the anti-inflammatory cytokine response in rheumatoid synovitis Rheumatology (Oxford) 2002 41 12 1341 1345 12468812 10.1093/rheumatology/41.12.1341

[B24] Prakken BJ van der Zee R Anderton SM Van Kooten PJ Kuis W Van Eden W Peptide-induced nasal tolerance for a mycobacterial heat shock protein 60 T cell epitope in rats suppresses both adjuvant arthritis and nonmicrobially induced experimental arthritis Proc Natl Acad Sci U S A 1997 94 7 3284 3289 9096385 10.1073/pnas.94.7.3284 PMC20361

[B25] Haque MA Yoshino S Inada S Nomaguchi H Tokunaga O Kohashi O Suppression of adjuvant arthritis in rats by induction of oral tolerance to mycobacterial 65-kDa heat shock protein Eur J Immunol 1996 26 11 2650 2656 8921951 10.1002/eji.1830261116

[B26] Lorenzo N Barbera A Dominguez MC Torres AM Hernandez MV Hernandez I Gil R Ancizar J Garay H Reyes O Altruda F Silengo L Padrón G Therapeutic effect of an altered peptide ligand derived from heat-shock protein 60 by suppressing of inflammatory cytokines secretion in two animal models of rheumatoid arthritis Autoimmunity 2012 45 6 449 459 22686732 10.3109/08916934.2012.697592

[B27] Wauben MH Immunological mechanisms involved in experimental peptide immunotherapy of T-cell-mediated diseases Crit Rev Immunol 2000 20 6 451 469 11396681

